# Transducer like proteins of *Campylobacter jejuni* 81-176: role in chemotaxis and colonization of the chicken gastrointestinal tract

**DOI:** 10.3389/fcimb.2015.00046

**Published:** 2015-05-27

**Authors:** Kshipra Chandrashekhar, Dharanesh Gangaiah, Ruby Pina-Mimbela, Issmat I. Kassem, Byeong H. Jeon, Gireesh Rajashekara

**Affiliations:** ^1^Food Animal Health Research Program, Department of Veterinary Preventive Medicine, Ohio Agricultural Research Development Center, The Ohio State UniversityWooster, OH, USA; ^2^Department of Environmental Health Sciences, School of Public Health, University of AlbertaEdmonton, AB, Canada

**Keywords:** chemotaxis, methyl accepting chemoreceptors, colonization, chicken, motility, organic acids, amino acids, virulence

## Abstract

Transducer Like Proteins (Tlps), also known as methyl accepting chemotaxis proteins (MCP), enable enteric pathogens to respond to changing nutrient levels in the environment by mediating taxis toward or away from specific chemoeffector molecules. Despite recent advances in the characterization of chemotaxis responses in *Campylobacter jejuni*, the impact of Tlps on the adaptation of this pathogen to disparate niches and hosts is not fully characterized. The latter is particularly evident in the case of *C. jejuni* 81-176, a strain that is known to be highly invasive. Furthermore, the cytoplasmic group C Tlps (Tlp5, 6, and 8) were not extensively evaluated. Here, we investigated the role of *C. jejuni* 81-176 Tlps in chemotaxis toward various substrates, biofilm formation, *in vitro* interaction with human intestinal cells, and chicken colonization. We found that the Δ*tlp6* and Δ*tlp10* mutants exhibited decreased chemotaxis toward aspartate, whereas the Δ*tlp6* mutant displayed a decreased chemotaxis toward Tri-Carboxylic Acid (TCA) cycle intermediates such as pyruvate, isocitrate, and succinate. Our findings also corroborated that more than one Tlp is involved in mediating chemotaxis toward the same nutrient. The deletion of *tlps* affected important phenotypes such as motility, biofilm formation, and invasion of human intestinal epithelial cells (INT-407). The Δ*tlp8* mutant displayed increased motility in soft agar and showed decreased biofilm formation. The Δ*tlp8* and Δ*tlp9* mutants were significantly defective in invasion in INT-407 cells. The Δ*tlp10* mutant was defective in colonization of the chicken proximal and distal gastrointestinal tract, while the Δ*tlp6* and Δ*tlp8* mutants showed reduced colonization of the duodenum and jejunum. Our results highlight the importance of Tlps in *C. jejuni*'s adaptation and pathobiology.

## Introduction

*Campylobacter jejuni* is a Gram-negative, thermophilic, and obligate microaerophilic bacterium that accounted for 7.5 million disability adjusted life years (DALYs) globally in 2010 (Murray et al., 2012). *C*. *jejuni* infects approximately 1 million people in the USA each year (Scallan et al., 2011). In 2012, 14.3 out of every 100,000 people were infected with *Campylobacter*, surpassing the 12.3 person goal set by the Centers for Disease Control and Prevention (CDC, 2013). These data highlight the need to control the incidence of food-borne *C. jejuni* outbreaks.

Chickens are a major source of *C. jejuni*, while a small proportion of campylobacteriosis cases can be attributed to other farm animals, and environmental sources (Wilson et al., 2008). However, the factors that contribute to successful colonization (in poultry) and virulence (in humans) remain not well defined. The ability to adapt to disparate environments is a key to *C. jejuni*'s diverse lifestyles in different hosts and its effective colonization of the gut (Hermans et al., 2011). These observations emphasize the need for a better understanding of the factors that contribute to *C. jejuni*'s adaptation.

Chemotaxis allows motile bacteria to travel toward a favorable niche or away from unfavorable conditions. Transducer Like Proteins (Tlp), also known as Methyl Accepting Chemotaxis Proteins (MCP), are chemoreceptors which mediate chemotaxis toward or away from chemoeffector molecules in an environment (Wadhams and Armitage, 2004). Although the basic components of chemotaxis are conserved among bacteria (Wadhams and Armitage, 2004), the number of Tlps/MCPs varies and has a weak correlation with bacterial genome size (Lacal et al., 2010). However, bacteria possessing genomes with higher number of Tlps such as *C. jejuni* are characterized by a complex lifestyle and a remarkable ability to interact with the host and other bacteria (Lacal et al., 2010). Notably, the genome sequence of *C. jejuni* NCTC11168 revealed homologs for ten putative chemotactic sensory receptors (Marchant et al., 2002). In *C. jejuni*, Tlps are classified into groups A, B and C, based on predicted domain structure and homology to chemoreceptors of other bacteria (Marchant et al., 2002). The group A Tlps (Tlp1, 2, 3, 4, 7, and 10) are integral membrane proteins with two transmembrane domains, a periplasmic ligand binding domain, and a conserved cytoplasmic signaling domain (Marchant et al., 2002). Group B Tlps [Tlp9 (CetA)] have two transmembrane domains and assist in energy taxis along with the signal sensing protein CetB (Marchant et al., 2002; Vegge et al., 2009). The group C Tlps (Tlp5, 6, and 8) possess cytoplasmic signaling domains but lack the transmembrane and periplasmic binding domain (Marchant et al., 2002). Previous research (e.g., Korolik and Ketley, 2008; Vegge et al., 2009; Tareen et al., 2010; Reuter and van Vliet, 2013; Li et al., 2014; Rahman et al., 2014) and the occurrence of multiple Tlps in the relatively small genome of *C. jejuni* strongly suggest that investigating these proteins might reveal crucial insights into the pathobiology of this pathogen.

*C. jejuni* has been shown to be chemotactic toward L-fucose, L-aspartate, L-cysteine, L-glutamate, L-serine, organic acid salts (pyruvate, succinate, fumarate, citrate, malate, and alpha-ketoglutarate), bile (from beef and chicken), and mucin (bovine gallbladder, and chicken and hog gastric mucin) (Hugdahl et al., 1988; Korolik and Ketley, 2008; Rahman et al., 2014). Nevertheless, our understanding of the role of Tlps in *C. jejuni* pathogenesis is still limited, especially with respect to the biological functions of the cytoplasmic group C Tlps. It is important to note that most of the previous studies on Tlps have been conducted using *C. jejuni* NCTC11168 or its variants (Table 1). For example, Tlp1, 3, 4, 7, 8, and 9 have been previously studied using *C. jejuni* NCTC11168 or NCTC11168-O (Vegge et al., 2009; Tareen et al., 2010; Reuter and van Vliet, 2013; Li et al., 2014; Rahman et al., 2014). However, the expression of *tlp* genes can vary based on growth conditions, isolation source, and strains (Day et al., 2012). Importantly, the overall contributions of Tlps to the success of highly invasive strains such as *C. jejuni* 81-176 have not received equal attention. *C. jejuni* 81-176 was originally isolated from the stool samples collected from a campylobacteriosis outbreak, which was associated with the consumption of raw milk (Korlath et al., 1985). *C. jejuni* 81-176 is known to have a relatively high invasion capacity in human epithelial intestinal cells *in vitro* (Hu and Kopecko, 1999), while this strain was experimentally shown to cause inflammatory diarrhea in human trials (Black et al., 1988). Although *C. jejuni* 81-176 and *C. jejuni* NCTC11168 are said to have the same complement of Tlp genes (Rahman et al., 2014), these strains are also known to have a marked difference in the genes encoding two Tlps. Specifically, *tlp3* has a naturally occurring mutation in *C. jejuni* 81-176 (Korolik and Ketley, 2008; Day et al., 2012), while *tlp7*, encoded by two genes (*cj0951c and cj0952*) in *C. jejuni* NCTC11168, is encoded by a single gene (*cjj81176-0975*) in *C. jejuni* 81-176 (Tareen et al., 2010). It was reported that the *tlp7* mutant was deficient in chemotaxis toward formic acid in *C. jejuni* B2 which, similar to NCTC11168, also has two genes encoding Tlp7 (Tareen et al., 2010). However, complementation using the corresponding gene from *C. jejuni* 81-176 restored the phenotype (Tareen et al., 2010). Subsequently, the functional significance of the difference in *tlp7* between the strains is not currently known. Furthermore, the impairment of *tlp3* in *C. jejuni* NCTC11168-O significantly reduced sodium deoxycholate-induced chemotaxis, colonization of jejunal mucosa in mice (Li et al., 2014), and motility and invasion of epithelial cell line, Caco-2 but had no significant impact on chicken colonization (Rahman et al., 2014). In another study, a *C. jejuni* NCTC11168 *tlp3* mutant also showed a decreased invasion of human Colo 205 epithelial cells and chicken embryo intestinal cells *in vitro* but the mutant's motility in a rich medium was not affected (Vegge et al., 2009). However, although *C. jejuni* 81-176 inconsistently and inefficiently colonized wild-type C3H mice (Chang and Miller, 2006), which is consistent with the aforementioned *tlp3* mutation, the strain is still capable of infecting humans and colonizing chickens (Black et al., 1988; Hendrixson and Dirita, 2004). Regardless, it is plausible that, in terms of specific tissue infection, high invasive capabilities might expose the pathogen and its Tlps to otherwise inaccessible niches and cognate substrates. Therefore, in this study, we investigated Tlps in the highly invasive *C. jejuni* 81-176 strain for their role in chemotaxis, biofilm formation, *in-vitro* interaction with human intestinal epithelial cells, and tissue specific colonization in the chicken host. For this purpose, we created isogenic mutants targeting all three Tlp groups, specifically *tlp4* and *tlp10* (Group A), *tlp9* (Group B), and *tlp6* and *tlp8* (Group C). A mutant targeting the last group C Tlp (*tlp5*) was not included, because we were unsuccessful in deleting the gene, which corroborated the observations of Hendrixson et al. (2001). Our data confirmed that more than one Tlp can mediate chemotaxis toward a particular substrate. Furthermore, certain Tlps contributed substantially to persistence (biofilm formation), interaction with human intestinal cells (INT 407), and chicken tissue colonization by *C. jejuni* 81-176.

**Table 1:** **Major and relevant findings on *C. jejuni* Tlps from previous studies**.

**Tlp protein**	**Protein designation**	**Ligand**	**Mutant phenotype**			**This study (*C. jejuni* 81-176)**
			**Motility**	***In vivo* colonization of chickens**	**Other**	
Tlp4 - 81-176 (Hendrixson and Dirita, 2004) - NCTC11168 (Vegge et al., 2009) -NCTC11168-O (Li et al., 2014)	Tlp4 (DocC)	- Sodium deoxycholate (Li et al., 2014) - No chemotaxis defect (Vegge et al., 2009)	- Decreased (Vegge et al., 2009)	- Decreased (Hendrixson and Dirita, 2004)	- Decreased invasion (Human Colo 205 cells, chicken embryo intestinal cells)	- No significant deficiency in the phenotypes was observed
Tlp5 - 81-176 (Hendrixson et al., 2001)	Tlp5	- Unknown	- Mutant could not be generated	- Mutant could not be generated	- Mutant could not be generated	- Mutant could not be generated
Tlp6 - 81-176 (Hendrixson and Dirita, 2004)	Tlp6	- Unknown	- Unknown	- No effect (cecum)	- Unknown	Decreased chemotaxis toward aspartate, isocitrate, succinate, and propionate - Decreased colonization of the chicken duodenum and jejunum
Tlp8 - NCTC11168 (Reuter and van Vliet, 2013)	CetZ	- Unknown (Energy taxis)	- No effect	No effect	- Opposing role in energy taxis	- Increased motility - Decreased biofilm formation - Decreased colonization of the chicken duodenum and jejunum - Decreased invasion in INT 407 cells
Tlp9 - NCTC11168 (Vegge et al., 2009; Reuter and van Vliet, 2013) - 81-176 (Hendrixson et al., 2001)	CetA	- Energy taxis - Decreased chemotaxis toward pyruvate	- Decreased (Vegge et al., 2009; Reuter and van Vliet, 2013) - Decreased in defined medium (Hendrixson et al., 2001)	- No effect	- Unknown	- Decreased invasion in INT 407 cells
Tlp10 - 81-176 (Hendrixson and Dirita, 2004) - NCTC11168 (Vegge et al., 2009)	DocB	- Unknown	- No effect	- Decreased in ceca and in proximal and distal intestinal tract	- Decreased invasion (NCTC11168 in Colo 205 cells, chicken embryo intestinal cells) (Vegge et al., 2009)	- Decreased chemotaxis toward aspartate and fumarate -Colonization defect in the chicken cecum, duodenum and jejunum

## Materials and methods

### Ethics statement

Animal experiments were conducted according to the guidelines of the Association for Assessment and Accreditation of Laboratory Animal Care International (AAALAC). The animal studies were approved by the Institutional Animal Care and Use Committee (IACUC), the Ohio State University. Chickens were housed at the Food Animal Health Research Program Animal Care Facility, which is fully accredited by AAALAC and the animals were supervised by a senior veterinarian. Infectious agents were administered using manual restraint for less than 1 min to minimize distress. Before necropsy, chickens were euthanized by carbon dioxide inhalation. This method is consistent with the recommendations of the panel on euthanasia of the American Veterinary Medical Association and by the Ohio State University Institutional Laboratory Animal Care and Use Committee.

### Bacterial strains, media, and growth conditions

Bacterial strains and plasmids used in this study are listed in Table S1. *C. jejuni* 81–176 (WT) was used to generate the *tlp* deletion mutants. *C. jejuni* strains were routinely grown on Mueller-Hinton agar (MH; Oxoid, Hampshire, United Kingdom) microaerobically [(85% N_2_ (v/v), 10% CO_2_ (v/v) and 5% O_2_ (v/v)] in a DG250 Microaerophilic Workstation (Microbiology International, Frederick, Maryland, USA) at 42°C. *E. coli* DH5α was used for cloning purposes and was routinely cultured using the Luria-Bertani (LB) medium at 37°C overnight. Growth media was supplemented with appropriate antibiotics, chloramphenicol (20 μg/ml for *E. coli*; 10 μg/ml for *Campylobacter*), kanamycin (30 μg/ml), and zeocin (50 μg/ml) where necessary.

### Targeted deletion of *tlp* genes

Deletion of target genes (*tlp4:cjj_0289; tlp6:cjj_0473; tlp8:cjj_1128; tlp9:cjj_1205; and tlp10: cjj_0046*) was achieved by double crossover homologous recombination using a suicide vector containing homologous sequences on either side of the respective *tlp* gene as described previously (Gangaiah et al., 2009; Rajashekara et al., 2009). Briefly, a 1 kb flanking region on either side of the target gene along with coding regions of the gene were amplified by PCR using the specific *tlp* F and R primers and *C. jejuni* 81–176 genomic DNA (Table S2). The PCR product was ligated into pZErO-1 to generate the plasmid pZErO-1-*tlp* using Fastlink™ DNA ligation kit (Epicentre, Madison). Inverse PCR was performed on pZErO1-*tlp* using the specific *tlp* INV F and INV R primers (Table S2) to amplify the plasmid without the majority of the *tlp* coding sequence; except for approximately 50 bp at the ends of the original *tlp* gene sequence. The kanamycin cassette from pUC4K was then cloned into the inverse PCR product to generate the suicide vectors pZErO-1-inv-*tlp*. The resulting suicide vectors were electroporated into *C. jejuni* 81–176 (Wilson et al., 2003). Recombinants were selected on MH agar plates containing the appropriate antibiotics. The deletion of the specific *tlp* gene was confirmed by PCR.

### Complementation of the Δ*tlp* mutants

The respective *tlp* coding sequences along with their potential promoter regions (~100–200 bp upstream of the target gene) were amplified by PCR using specific primers and cloned into pRY111, an *E. coli-Campylobacter* shuttle vector (Yao et al., 1993) (Table S2). Since potential promoter regions for *tlp6* and *tlp10* are located further upstream, sequence encoding the open reading frame (ORF) with the ribosomal binding site was cloned into pRY111 followed by directional cloning of the potential promoter region upstream of the ORF [For *tlp6* (*cjj_0473*): intergenic region between *cjj_0474* and *rpmB*; for *tlp10 (cjj_0046)*: region between *ccpA (cjj_0047)* and *cjj_0048*]. The recombinant plasmids were then transformed into *E. coli* DH5α, purified, and subjected to restriction analysis to confirm that they carried the desired sequence. The plasmids were introduced into the appropriate *tlp* mutant by biparental conjugation as described previously (Miller et al., 2000). Transconjugants were selected on MH agar plates containing kanamycin and chloramphenicol and one transconjugant for each target was selected, further confirmed by PCR to verify the presence of WT copy of the cognate gene, designated as *tlp* comp, and used in complementation studies.

### Syringe capillary assay for chemotaxis

To quantify chemotaxis, we adapted a modified capillary chemotaxis assay that quantitatively measured bacterial tactic responses (Figure S1). Capillary assays are the most commonly used methods to assess bacterial chemotaxis (Lewus and Ford, 2001). The assay was previously used for quantifying chemotaxis in subsurface microaerophilic bacteria (Mazumder et al., 1999) and other *Epsilonproteobacteria*, namely *Helicobacter pylori* (Cerda et al., 2003, 2011). Bacterial culture from each *C. jejuni* strain grown microaerobically at 42°C for 18 h on MH agar were harvested using a sterile disposable loop, washed and resuspended by gentle pipetting in chemotaxis buffer (Phosphate Buffered Saline, pH 7.4), and OD_600_ was adjusted to 0.5. A 100 μl of a solution containing 100 mM of the compound to be tested for chemotactic response (buffer alone served as a control) was aspirated through a 22 G stainless-steel needle (0.25 mm diameter × 20 mm long) into a 1 ml tuberculin syringe. The concentration of the compound was selected based on previous studies and a series of preliminary experiments that showed that 100 mM resulted in the strongest chemotaxis response (Vegge et al., 2009; Tareen et al., 2010). A 100 μl of the adjusted bacterial suspension was drawn into a 200 μl disposable pipette tip, which was then sealed from one end. The needle-syringe system was fitted to a pipette tip in such a way that most of the needle immersed into the bacterial suspension. The system was positioned horizontally and incubated at 42°C for 1 h. Finally, the needle-syringe system was detached and bacterial suspension in the syringe was 10-fold serially diluted in the chemotaxis buffer. Dilutions were plated onto MH agar plates, incubated for 24 h at 42°C microaerobically, and the CFU were counted. Each assay was performed in triplicate. Results were expressed as the mean of three independent assays. To express the taxis toward a test compound, Relative Chemotaxis Ratio (RCR) was calculated as the ratio between the numbers of bacteria entering the test needle-syringes and those in the control (buffer only) needle-syringes. A test compound was considered as an attractant if the RCR was significantly = 2 (*P* < 0.05) (Adler, 1973; Moulton and Montie, 1979; Mazumder et al., 1999; Cerda et al., 2003). A mutant was considered deficient in chemotaxis toward a substrate if both the corresponding RCR-value was significantly < 2 (*P* < 0.05) and the CFU of the mutants were significantly lower (*P* < 0.05) than those of the wildtype. A *C. jejuni* 81-176 *cheY* mutant which is incapable of directional movement (negative control; Yao et al., 1997) and 0.1% porcine gastric mucin (positive control; Sigma) were also used to evaluate the integrity of the assay. To test the response to repellents, *C. jejuni* cultures were mixed with a repellent and the bacteria that entered the syringe, which in this instance contained only buffer, to escape the repellent were quantified as described above. To account for any methodological bias, capillary chemotaxis results were further verified by using the disc method (Vegge et al., 2009; Tareen et al., 2010) for selected compounds.

### Motility and biofilm assays

To determine motility, *C. jejuni* mutants and the wild type (WT) were grown microaerobically at 42°C for 18–24 h on MH agar and resuspended in MH broth to an OD_600_ of 0.05. Bacterial cells were stabbed (2 μl) with pipette tips into semisolid MH agar (0.4% agar) plates. Plates were incubated for 48 h at 42°C microaerobically. Motility was assessed by measuring the diameter of the zone of motility after 48 h (Rajashekara et al., 2009). A *C. jejuni* 81-176 *cheY* mutant which is deficient in motility and the RY213 strain (diploid for *cheY*) which exhibits increased motility (Yao et al., 1997) were used as controls.

Static biofilm formation was assessed in borosilicate tubes by incubating 1 ml of 0.05 OD_600_ adjusted MH broth culture at 42°C microaerobically for 72 h without shaking (Gangaiah et al., 2009). The biofilms were stained with 1% crystal violet for 15 min, washed to remove excess stain, and quantified by measuring the absorbance (OD_570_) after incubation in 1 ml DMSO (80%) for 48 h.

### Quantitative reverse transcriptase PCR (qRT-PCR) of biofilm associated genes

The *C. jejuni* WT and Δ*tlp8* strains were examined for changes in expression of biofilm related genes in 3 day old biofilms and overnight grown planktonic shaking cultures. The cultures were adjusted to an initial OD_600_ of 0.05 in MH broth and incubated at 42°C microaerobically either in standing condition for 3 days (biofilm growth) or overnight under shaking at 100 rpm (planktonic). Total RNA was extracted using RNeasy Mini Kit (Qiagen) and cDNA was synthesized using SuperScript® III First-Strand Synthesis SuperMix (Invitrogen). RNA and cDNA concentrations and purity were determined using NanoDrop ND-2000c spectrophotometer (Wilmington, DE).

qRT-PCR analysis was performed on genes involved in biofilm formation. These included: *kpsM*, capsular polysaccharide synthesis; *pglH*, protein glycosylation; *neuB*, lipo-oligosaccharide; *fliS*, flagella protein synthesis; *cj0688*, phosphate acetyltransferase; *maf5*, flagella formation and *luxS*, quorum sensing (Joshua et al., 2006; Reeser et al., 2007). Gene specific primers (Table S2) used in this analysis have been described previously (Drozd et al., 2014). qRT-PCR was performed using SensiMixPlus® SYBR RT-PCR Kit (Quantace, Norwood, MA) in a Realplex^2^ Mastercycler (Eppendorf, Westbury, NY). Nuclease free water (no DNA) was used as negative control. The relative levels of expression of target genes were normalized to 16S rRNA gene expression of the same strain. The relative fold changes in gene expression was calculated using the comparative threshold cycle (CT) method to yield fold-difference in transcript level compared to WT (Livak and Schmittgen, 2001). The qRT-PCR was performed three times with duplicate samples in each assay.

### Adherence and invasion assay

As described in Kassem et al. (2012) and Gaynor et al. (2005), each well of a 24-well tissue culture plate was seeded with 1.4 × 10^5^ INT 407 cells (human embryonic intestine cells, ATCC CCL 6) in MEM with 10% (v/v) fetal bovine serum (FBS) and incubated for 18 h at 37°C under 5% CO_2_. Following incubation, cells were infected with *C. jejuni* WT and mutants at a multiplicity of infection 100:1 in both adherence and invasion assays. To assay for adherence, the infected monolayers were incubated for 3 h followed by washing the INT 407 cells 3 times with MEM. Cells were then lysed using 0.1% (v/v) Triton X-100, serially diluted (10-fold) in MEM and 100 μl of each dilution were spread on MH agar plates. The plates were incubated for 48 h at 42°C under microaerobic conditions after which CFU were counted. To assess invasion, INT 407 cells were infected for 3 h at 37°C after which cells were treated with gentamicin (150 μg/ml) and incubated for an additional 2 h. Subsequently, infected cells were rinsed with MEM three times, lysed with 0.1% Triton-X 100, serially diluted in MEM, and spread on MH agar plates to determine the number of CFU. In parallel, we also cultured the supernatant of gentamicin treated monolayers to ensure the quality of the gentamicin protection assay.

### Chicken colonization studies

Three day-old specific pathogen free chicks (*n* = 6/group) from our hatching facility (Food Animal Health Research Program, OARDC, Wooster, OH) were used in this study. Birds in each group were housed in a separate cage, and the birds were further confirmed to be *Campylobacter* free by testing cloacal samples. The birds were then inoculated orally with 10^4^ CFU of the *C. jejuni* WT and Δ*tlp6*, 8, 9, and *10* mutants in 200 μl of 1X PBS (pH 7.4). Chicks were euthanized 7 days post-inoculation. The ceca, duodenum, jejunum, liver, spleen, and bursa were collected aseptically, weighed, homogenized, serially diluted in 1X PBS (pH 7.4), and 100 μl of the homogenates were spread onto MH agar containing *Campylobacter* selective supplement with or without kanamycin. Plates were incubated at 42°C microaerobically and CFU/gram of tissues was determined.

### Statistical analysis

Data analysis was performed using One-Way analysis of variance (ANOVA) with Tukey's post-test. Statistical significance of data was also determined using Student's *t*-test in cases where only two data sets were compared. Data from the chicken colonization experiment were analyzed using the Mann-Whitney test. *P* = 0.05 (α level) was considered statistically significant.

## Results

### Contribution of *tlp*s to chemotaxis in response to amino acids and organic acids

**Figure 1:**
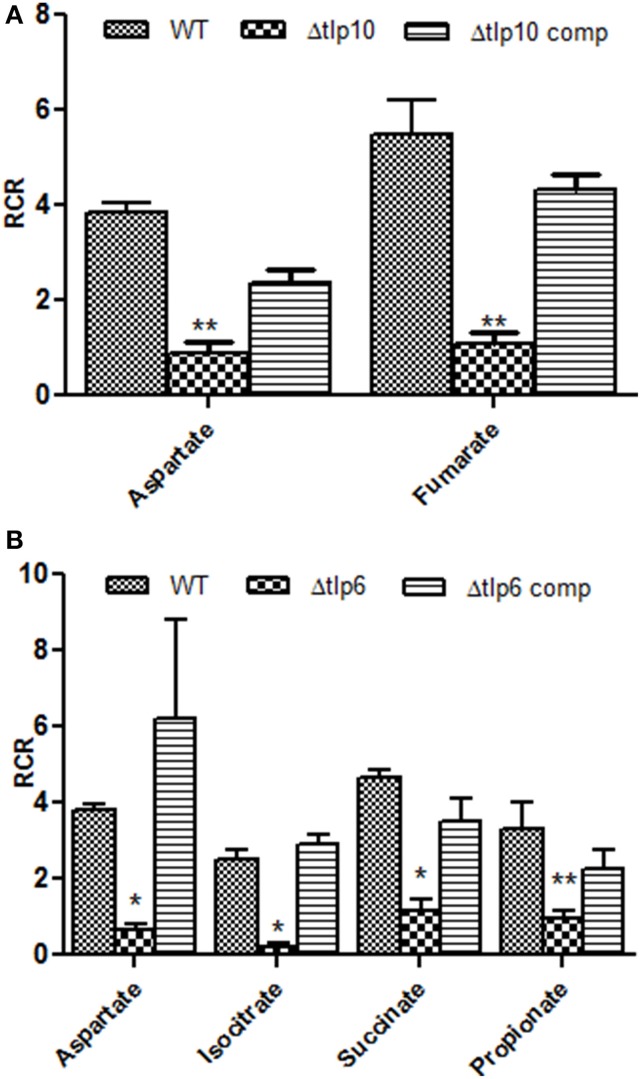
**The Δ***tlp*** mutants that are defective in chemotaxis toward essential nutrients**. Chemotaxis of *tlp10* **(A)** and *tlp6* **(B)** was assessed by determining the relative chemotactic ratio (RCR) which is the ratio of bacterial numbers migrating toward the chemical in the syringe to the bacterial numbers migrating toward the chemotaxis buffer. Chemotaxis was determined using the capillary method and significant results were further confirmed using the disc diffusion method (Table S3). Mucin and a Δ*cheY* mutant were used as a positive and negative control, respectively. An RCR-value of 2 or above indicates chemotaxis toward the test chemical. Only those Δ*tlp* mutants with a significant defect in chemotaxis are shown and the complete chemotaxis results for all compounds tested are listed in Table 2. The data represent the average and SE of three experiments with 3 replicates in each experiment. ^*^P ≤ 0.05, ^**^P ≤ 0.01.

To determine the contribution of Tlps toward chemotaxis, we generated deletion mutants of *tlp4, 6, 8, 9*, and *10*. The mutants did not exhibit a growth defect while incubated in MH broth (data not shown). Our preliminary analysis indicated that RCR-values for WT *C. jejuni* toward known chemoattractants were >2 and for chemorepellants such as bile acids (cholic acid and deoxycholic acid) was < 0.1 (Hugdahl et al., 1988; Cerda et al., 2003), which further validated the usefulness of the syringe-capillary method to assess chemotaxis in *C. jejuni*. Furthermore, using the capillary assay we show that *C. jejuni* 81-176 exhibited strong chemotaxis toward 0.1% porcine gastric mucin (RCR = 9.0), while a *cheY* mutant (non-motile) had an RCR below the detection limit (~ 0), because no or very few colonies were retrieved from the syringe containing tested chemoattractants (aspartate, fumarate, propionate, and pyruvate). All compounds tested in this chemotaxis assay were used at a concentration of 100 mM which was determined based on our preliminary studies using varying concentrations of compounds from 10 to 100 mM (Figure S2A). Figure 1 shows *tlp* mutants with significantly defective chemotaxis against various substrates tested in this study. Chemotactic responses of *tlp* mutants to various substrates are shown in Table 2. Furthermore, the response of the mutants to chemorepellents (Cholic acid and Deoxycholic acid) was not significantly different compared to those of the wildtype. Specifically, all strains exhibited an RCR of <0.1 in response to the repellents.

**Table 2:** **RCR-values for the WT and chemotaxis mutants for all compounds tested**.

**Compound**	**WT**	**Δ*tlp4***	**Δ*tlp6***	**Δ*tlp8***	**Δ*tlp9***	**Δ*tlp10***
	**Average CFU**	**Average CFU**	**Average CFU**	**Average CFU**	**Average CFU**	**Average CFU**
	**(RCR)^a^**	**(RCR)**	**(RCR)**	**(RCR)**	**(RCR)**	**(RCR)**
Aspartate	(9.76 ± 0.85)^*^10^5^ (3.81 ± 0.3)	(2.21 ± 0.63)^*^10^6^ (8.65 ± 2.46)	(1.74 ± 0.59)^*^10^5^ (**0.68 ± 0.23**)	(6.17 ± 1.56)^*^10^5^ (2.41 ± 0.61)	(8.69 ± 3.33)^*^10^5^ (3.39 ± 1.3)	(2.30± 1.05)^*^10^5^ (**0.90 ± 0.41**)
L-glutamine	(3.79 ±0.48)^*^10^6^ (4.19 ± 0.5)	(1.26 ± 0.14)^*^10^7^ (13.98 ± 1.48)	(1.64 ± 0.44)^*^10^6^ (1.81 ± 0.48)	(3.13 ± 0.84)^*^10^6^ (3.46 ± 0.93)	(3.3 ± 0.78)^*^10^6^ (3.65 ± 0.87)	(1.56 ± 0.17)^*^10^6^ (1.73 ± 0.19)
L-serine	(1.21± 0.23)^*^10^6^ (4.03 ± 0.76)	(2.29 ± 0.23)^*^10^6^ (7.62 ± 0.75)	(1.6± 0.64)^*^10^6^ (5.32 ± 2.12)	(1.33 ± 0.36)^*^10^6^ (4.42 ± 1.21)	(1.15 ± 0.32)^*^10^6^ (3.82 ± 1.08)	(1.19 ± 0.52)^*^10^6^ (3.95 ± 1.73)
Fumarate	(2.47 ±0.58)^*^10^6^ (5.48 ± 1.28)	(4.64 ± 0.58)^*^10^6^ (10.32 ± 1.29)	(1.44 ± 0.34)^*^10^6^ (3.19 ± 0.76)	(1.31 ± 0.14)^*^10^6^ (2.90 ± 0.31)	(1.89 ± 0.65)^*^10^6^ (4.21 ± 1.44)	(5.09 ± 2.16)^*^10^5^ (**1.13 ± 0.48**)
Isocitrate	(7.05 ± 1.15)^*^10^5^ (2.51 ± 0.41)	(1.17 ± 0.64)^*^10^6^ (4.17 ± 2.28)	(6.18 ± 3.65)^*^10^4^ (**0.22 ± 0.13**)	(1.43 ± 0.45)^*^10^6^ (5.08 ± 1.59)	(1.59 ± 0.33)^*^10^6^ (5.65 ± 1.18)	(7.56 ± 2.05) ^*^10^5^ (2.69 ± 0.73)
Formate	(6.02 ±0.18)^*^10^6^ (4.41 ± 0.13)	(8.43 ± 2.57)^*^10^6^ (6.18 ± 1.88)	(3.29 ± 1.24)^*^10^6^ (2.41 ± 0.91)	(3.11 ± 0.93)^*^10^6^ (2.28 ± 0.68)	(6.55 ± 1.66)^*^10^6^ (4.8 ± 1.22)	(2.32 ± 0.34)^*^10^6^ (1.7 ± 0.25)
Succinate	(1.95 ±0.13)^*^10^6^ (4.68 ± 0.32)	(3.68 ± 2.32)^*^10^6^ (8.81 ± 5.56)	(4.88 ± 1.96)^*^10^5^ (**1.17 ± 0.47**)	(2.29 ± 0.65)^*^10^6^ (5.48 ± 1.56)	(2.14 ± 1.75)^*^10^6^ (5.13 ± 4.18)	(7.81 ± 2.51)^*^10^5^ (1.87 ± 0.60)
Pyruvate	(1.85 ± 0.04)^*^10^6^ (3.46 ± 0.08)	(2.95 ± 0.21)^*^10^6^ (5.52 ± 0.39)	(1.07± 0.18)^*^10^6^ (2.01 ± 0.32)	(1.13± 0.34)^*^10^6^ (2.11 ± 0.63)	(6.95 ± 0.59)^*^10^5^ (1.3 ± 0.11)	(1.27 ± 0.3)^*^10^6^ (2.37 ± 0.55)
Propionate	(4.04 ± 1.47)^*^10^6^ (3.33 ± 1.21)	(3.86 ± 0.17)^*^10^6^ (3.19 ± 0.14)	(1.14 ± 0.45)^*^10^6^ (**0.94 ± 0.37**)	(6.8 ± 2.33)^*^10^6^ (5.6 ± 1.92)	(6.03 ± 1.15)^*^10^6^ (4.97 ± 0.95)	(4.38 ± 3.64)^*^10^6^ (3.61±3)

a *The RCR was calculated by taking the ratio of bacterial numbers migrating toward the chemical in the syringe to the bacterial numbers migrating toward the chemotaxis buffer. The results show the means of three independent assays. An RCR-value of 2 and above indicates chemotaxis toward the test chemical (Mazumder et al., 1999). RCR-values that were significantly lower than 2 (P < 0.05) are indicated in bold font*.

Based on the RCR indices and CFU numbers, the Δ*tlp6* and Δ*tlp10* mutants were defective in chemotaxis toward aspartate compared to WT (Figures 1A,B). The Δ*tlp10* mutant showed decreased chemotaxis toward fumarate, while the Δ*tlp6* mutant showed a diminished chemotaxis toward succinate, isocitrate, and propionate (Figure 1B). Chemotaxis defects observed in the mutants was restored to WT levels in the complemented strains; except in Δ*tlp10* where there was partial complementation for chemotaxis to aspartate (Figure 1). However, this partial complementation observed in case of *tlp10* was not due to a polar effect, because the relative expression of a downstream gene (*cjj_0045*) was similar to the WT (ΔΔCt fold change for *cjj_*0045 was 1.068). Chemotaxis results observed above for the Δ*tlp* mutants were further confirmed by assessing chemotaxis using the disc method for selected substrates (Table S3).

### Contribution of *tlps* towards motility and biofilm formation

**Figure 2:**
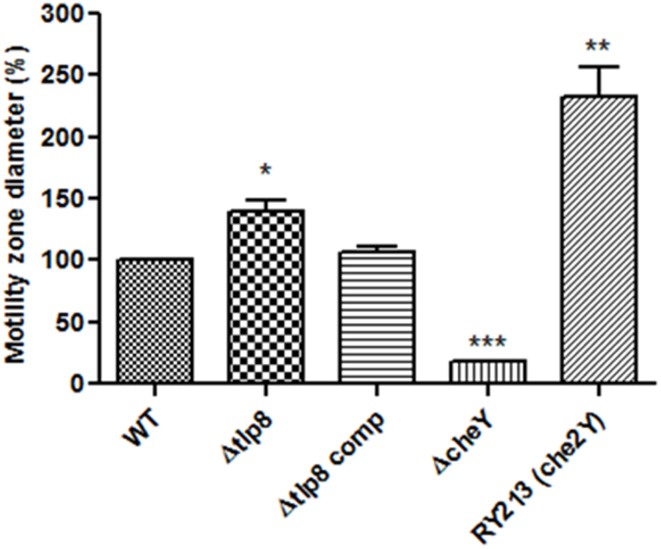
**Role of chemoreceptors in motility**. Histogram showing the diameter of the zone of motility expressed in percentages relative to the WT. The zone of motility was determined after stabbing 2 μL of *C. jejuni* (OD_600_ of 0.05) in the middle of a semi-solid (0.4%) MH agar plate. The agar plates were incubated microaerobically for 48 h and motility was assessed by measuring the diameter of the zone of motility. The Δ*tlp8* mutant showed increased motility. Motility was restored to values similar to the WT levels in the complemented strain. A *C. jejuni* 81-176 *cheY* mutant and the RY213 strain (diploid for *cheY*) were used as controls. ^*^P < 0.05, ^**^P < 0.01, ^***^P < 0.001.

**Figure 3:**
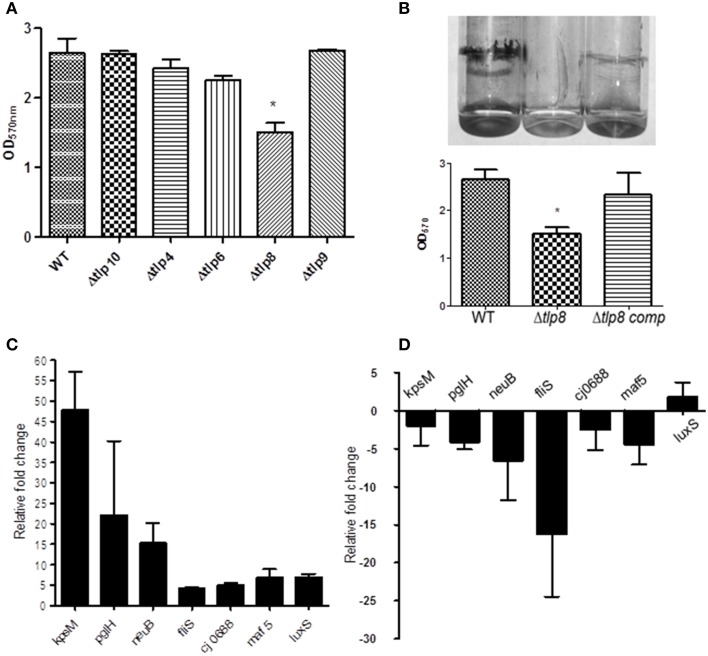
**Contribution of *tlps* to biofilm formation. (A)** Biofilm was assessed by crystal violet staining. One ml of *C. jejuni* cultures (OD_600_ of 0.05) was incubated in borosilicate tubes microaerobically for 72 h, the tubes were washed with distilled water and stained using (1%) crystal violet. Extra stain was removed by washing and the biofilm were suspended in 1 mL DMSO (80%) for quantification by a spectrophotometer. Each bar represents the mean ± SE of 3 independent experiments. ^*^P ≤ 0.05. (**B)** The Δ*tlp8* mutant shows decreased biofilm formation and this defect was restored in the complemented strain. Each bar represents the mean ± SE of 3 independent experiments, ^*^P ≤ 0.05. **(C,D)** Transcriptional changes in biofilm associated genes in the Δ*tlp8* mutant measured by qRT-PCR using 3 day old biofilm cultures **(C)** and overnight grown planktonic culture **(D)**. The difference in gene expression relative to the WT was determined by the threshold cycle (*CT*) method. The assay was repeated three times. The data represent the mean relative fold change in expression ± SE.

Most of the Δ*tlp* mutants demonstrated either a slight or no defect in motility compared to the WT strain. However, the Δ*tlp9* mutant showed the most significant defect (a 54.4% decrease, *P* = 0.0131) (Figure S2B). The Δ*tlp8* (Δ*cetZ*) mutant exhibited an increased motility phenotype (Figure 2). Under bright field microscopy, no visible differences were observed in the motility of Δ*tlp8* mutant compared to the WT (data not shown). However, the Δ*tlp8* mutant revealed a defect in biofilm formation under microaerobic conditions (Figures 3A,B). To further investigate this observation, we analyzed the expression of genes that have been previously implicated in biofilm formation in the Δ*tlp8* biofilm and planktonic cultures. The *kpsM, pglH, neuB, fliS, maf5*, and *cj0688* genes were upregulated in 3 day old Δ*tlp8* biofilms, but they were downregulated in its planktonic culture. However, the *luxS* gene was upregulated in the Δ*tlp8* mutant in both the biofilm and the planktonic phases (Figures 3C,D).

### Role of *tlp*s in adhesion to and invasion of INT 407 cells

**Figure 4:**
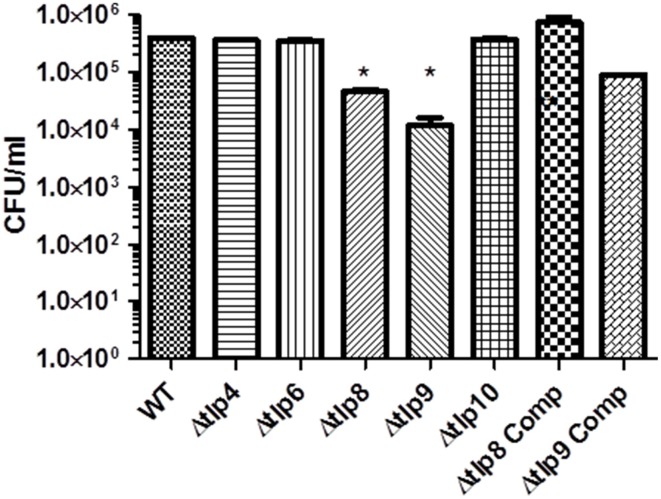
**Contribution of *tlps* to Invasion of INT 407 cells**. Invasion was assessed using the gentamicin protection assay. INT 407 cells were infected with *C. jejuni* strains for 3 h after which the cells were treated with gentamicin (150 μg/ml) and incubated for an additional 2 h. The cells were then washed and lysed and the resulting lysate was diluted and spread (100 μL) on MH agar plates. The Δ*tlp8* and *9* mutants displayed an invasion defect in INT 407 human intestinal epithelial cells. The data represent the average of 2 experiments with 3 replicates in each experiment. ^*^P ≤ 0.05.

To investigate if Tlps affect the interaction with human cells, we examined the Δ*tlp* mutants' adherence to and invasion of INT 407 human intestinal epithelial cells. In comparison to WT, there were no significant differences (*P* > 0.05) in the mutants' CFU numbers that adhered to the INT 407 cells (Figure S3). The Δ*tlp8* and the Δ*tlp9* mutants exhibited a significant (*P* ≤ 0.05) defect in invasion of INT 407 cells (Figure 4) with 1–2 logs less bacteria recovered compared to WT, respectively.

### Contribution of *tlp*s to organ specific colonization in chicken

**Figure 5:**
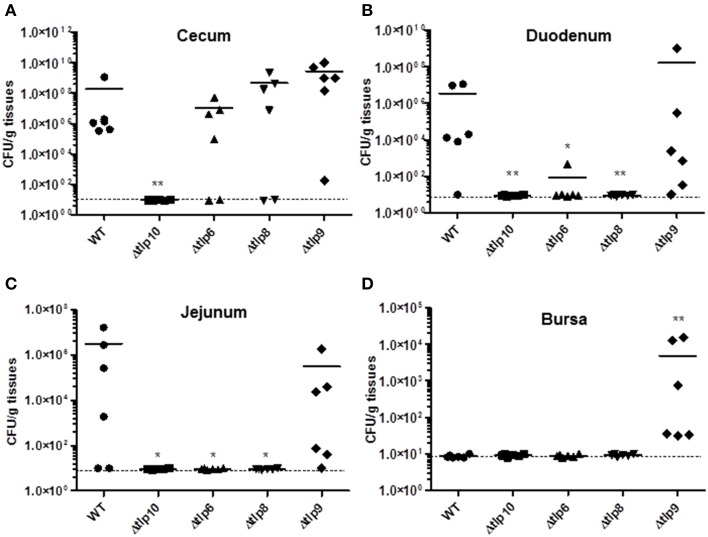
**Colonization of the Δ***tlp*** mutants in chickens**. Three day-old chicks (*n* = 6/group) were inoculated orally with 10^4^ CFU of the *C. jejuni* WT and Δ*tlp6, 8, 9*, and *10* mutants. Seven days post-inoculation, the ceca, duodenum, jejunum, liver, spleen, and bursa were collected, homogenized, and 100 μL of serially diluted homogenates were spread onto MH agar containing *Campylobacter* selective supplement. (**A)** The Δ*tlp10* mutant exhibited a significant defect in colonization of the cecum. (**B)** The Δ*tlp8* and *10* mutants exhibited a colonization defect in the duodenum. **(C)** The Δ*tlp6*, 8, and 10 mutants showed a decreased colonization in the jejunum. (**D**) The Δ*tlp9* mutant exhibited an increased colonization of the bursa. Each data point represents CFU/g of tissue, ^*^P < 0.05, ^**^P ≤ 0.01. Dotted line represents the detection limit. The horizontal bars represent the arithmetic mean values for CFU.

We investigated the *in-vivo* colonization potential of the Δ*tlp* mutants that showed a significant defect in the phenotypes listed in the studies above. We determined the role of these *tlp*s in organ specific colonization in the cecum, jejunum, duodenum, liver, spleen, and bursa. The Δ*tlp10* mutant was severely defective in colonization of the cecum, jejunum, and duodenum (Figures 5A–C) with no bacteria recovered from any of these tissues. The Δ*tlp6* mutant showed a 1 log decrease in cecal colonization compared to WT. Colonization potential was also affected in the Δ*tlp6* and *8* mutants in duodenum (2–7 log less) and jejunum (5–7 log less) compared to WT. Interestingly, only the Δ*tlp9* mutant was able to colonize all the examined segments of the intestine. The liver and spleen showed no colonization by *C. jejuni* WT or the *tlp* mutants. However, bursal colonization was observed only with the Δ*tlp9* mutant; even as no bacteria were recovered from the bursa of the WT inoculated group (Figure 5D).

## Discussion

In this study, we investigated the contribution of five chemoreceptors (belonging to the three major Tlp groups A, B, and C) toward the chemotaxis of *C. jejuni* 81-176, motility, biofilm formation, invasion of human epithelial intestinal cells, and colonization of the chicken host. A previous study identified Tlp1 as the aspartate chemosensory receptor in *C. jejuni* (Hartley-Tassell et al., 2010). Here, we observed a decreased chemotaxis toward aspartate and glutamate in the Δ*tlp6* and Δ*tlp10* mutants. This corroborated that more than one chemoreceptor can mediate chemotaxis toward a particular substrate (Rahman et al., 2014). This is not uncommon in bacterial chemotaxis where heterologous receptors can generate a cooperative signaling behavior similar to that observed for the chemoreceptors of *Sinorhizobium meliloti* and *E. coli* (Meier et al., 2007; Raterman and Welch, 2013). More importantly, the association of *tlp6* and *tlp10* with chemotaxis toward aspartate has not been reported before. It can be thus hypothesized that the ability of more than one Tlp to sense the same ligand provides robustness to the *C. jejuni* chemotaxis system, which is a vital factor in the adaptation of this enteric pathogen.

Our study also provides analysis on the role of the tested *tlps* in chemotaxis toward physiologically relevant organic acids. The Δ*tlp6* and 10 mutants showed decreased chemotaxis toward organic acids (aspartate, isocitrate, succinate, propionate, and fumarate); many of which are TCA cycle intermediates (Figure 1). Interestingly, Tlp6 is a group C cytosolic chemoreceptor, and the Δ*tlp6* mutant showed chemotaxis defect toward isocitrate, propionate, and succinate. Previous studies of cytosolic chemoreceptors in *Rhodobacter sphaeroides, S. meliloti*, and *H. pylori* (Wadhams et al., 2002; Meier et al., 2007; Schweinitzer et al., 2008) suggest that intracellular Tlps modulate bacterial chemotaxis toward environments with optimum concentration of metabolites which affect the energy status of the bacteria (Porter et al., 2011). The latter confers an obvious competitive advantage and further highlight the importance of Tlps in the survival strategies of *C. jejuni*. It was notable that the RCR of certain mutants (e.g., the Δ*tlp4* mutant) was higher than that of the WT in response to some substrates (Table 2). While this may seem counterintuitive, it can be explained by the evolutionary pressure to seek a large variety of substrates via interaction of different Tlps to enhance adaptation and competitiveness (Sourjik and Armitage, 2010). In that regard, the absence of one Tlp from a Tlp cooperative cluster might have enhanced the interactions of the others, subsequently enhancing chemotaxis to certain substrates.

Studies on *C. jejuni* NCTC11168 have shown that the *tlp1, tlp3, docB*, and *docC* mutants displayed a decreased ability to invade human intestinal epithelial and chicken embryo cells (Vegge et al., 2009; Tareen et al., 2010). In this study, *tlp8* and *tlp9* were the only mutants defective in invasion of INT407 cells. Tlp8 (CetZ) and Tlp9 (CetA) are involved in energy taxis (Reuter and van Vliet, 2013), which emphasizes the importance of these two genes and the cognate acquisition of energy in virulence. Interestingly, the Δ*tlp8* mutant was defective in biofilm formation under microaerophilic conditions despite exhibiting an increased motility on semi-solid agar plates. Previously, it has been shown that a *C. jejuni Cj1496c* hypermotile mutant was defective in colonization of the chicken host (Kakuda and Dirita, 2006). It was suggested that the deletion of *Cj1496c* may lead to elevated levels of phosphorylated CheY, which increased the tumbling frequency. Furthermore, a diploid *cheY* mutant displayed non-adherent, non-invasive, and hypermotile phenotypes (Yao et al., 1997) similar to what was observed with the Δ*Cj1496c* mutant. However, the mechanisms involved in the *cheY*-diploid phenotype have not been delineated. Similarly, the decreased invasiveness of the Δ*tlp8* mutant suggests an involvement of other flagella independent mechanisms which require further investigation. It is possible that to meet crucial energy needs in the Δ*tlp8* mutant, compensatory mechanisms such as increased motility might favor randomly detecting energy sources.

Biofilm formation is a complex process that is affected by quorum sensing, flagella, aerobic stress, and nutrient availability in *C. jejuni* (Reeser et al., 2007; Reuter et al., 2010). Additionally, biofilm formation in *C. jejuni* has been shown to involve both flagella dependent and independent mechanisms (Kalmokoff et al., 2006; Reuter et al., 2010). Interestingly, BLAST analysis of Tlp8 revealed a 41% identity with BdlA protein of *Pseudomonas aeruginosa*. BdlA is a putative MCP which mediates biofilm dispersion in *P. aeruginosa*, a motility independent phenotype (Morgan et al., 2006). However, unlike BdlA, Tlp8 doesn't contribute to biofilm shedding (Figure S4). Furthermore, biofilm-related genes (*maf5, kpsM, fliS, neuB*, and *cj0688*) (Joshua et al., 2006) were upregulated in the Δ*tlp8* mutant in biofilms as compared to planktonic culture (Figure 3D). These results likely suggest that Tlp8 may be interacting with other factors which dictate biofilm formation, highlighting potential complex interactions between Tlps, nutrient sensing, motility, and biofilm formation in *C. jejuni*.

We observed that *tlps* contribute to differential colonization of *C. jejuni* in different sections of the chicken gastrointestinal tract (cecum, jejunum, and duodenum). Consistent with previous reports (Hendrixson and Dirita, 2004) (Table 1), the Δ*tlp10* (Δ*docB*) mutant was highly defective in colonization of the cecum and the Δ*tlp9* (Δ*cetA*) mutant showed no colonization defect. The Δ*tlp6* and *10* mutants colonized differently in the duodenum and the jejunum, which also coincided with defects in the chemotaxis of these mutants toward essential nutrients such as aspartate, pyruvate, and fumarate. These observations further suggest that a relationship between substrate-specific chemotaxis and tissue-specific colonization may exist and further investigation of these interactions may provide a better insight into *C. jejuni*-host interactions.

In comparison with other studies (Table1), our Δ*tlp4* and Δ*tlp8* mutants exhibited slightly different phenotypes. Specifically, our Δ*tlp4* mutant was not deficient in the phenotypes that we tested. However, a *tlp4* mutant in *C. jejuni* NCTC11168 exhibited decreased motility and reduced invasion of human Colo 205 epithelial cells and chicken embryo intestinal cells *in vitro* (Vegge et al., 2009). Furthermore, a *tlp4* mutant in *C. jejuni* NCTC11168-O was deficient in chemotaxis in response to sodium deoxycholate (Li et al., 2014). Hendrixson and Dirita (2004) showed that a *tlp4* mutant in *C. jejuni* 81-176 exhibited decreased colonization of the chicken cecum; however, this was not tested in our study. Finally, a Δ*tlp8* mutant in NCTC 11168 did not exhibit increased motility (Reuter and van Vliet, 2013). While the reasons behind these discrepancies are not currently clear and might be due to the use of different testing methods, it is known that the expression of *tlp* genes can vary based on growth conditions, isolation source, and strains (Day et al., 2012). It is also important to note that Tlp4 and Tlp8 in *C. jejuni* 81-176 exhibit 100 and 99% protein sequence identity with their cognate homologs in *C. jejuni* NCTC-11168, respectively. Furthermore, it is possible that certain Tlps might confer different phenotypes to *C. jejuni* 81-176, which conforms to our initial predictions. In turn, the invasive properties of *C. jejuni* 81-176 might provide access to niches and cognate substrates that may not be available to *C. jejuni* NCTC-11168.

In conclusion, we delineated the contributions of Tlps and cognate chemotaxis to survival and host colonization phenotypes of *C. jejuni* 81-176. The effects on biofilm formation, motility, and host colonization highlight the far reaching implications of impairing the chemotaxis system in *C. jejuni*, a pathogen that preferentially relies on non-sugar carbon sources for energy production, adaptation, and success in disparate niches. Our data also emphasize the complex interactions that allow a bacterial pathogen to respond to its environment.

### Conflict of interest statement

The authors declare that the research was conducted in the absence of any commercial or financial relationships that could be construed as a potential conflict of interest.
