# Supplementary material for: Transducer like proteins of Campylobacter jejuni 81-176: role in chemotaxis and colonization of the chicken gastrointestinal tract
Source: Front Cell Infect Microbiol. 2015 May 27;5:46. doi: 10.3389/fcimb.2015.00046 (PMC4444964; doi:10.3389/fcimb.2015.00046)
Supplement: Supplementary file 1 [file Table1.DOCX]

**Table S1: Bacterial strains and plasmids used in this study.**

| **Strains** | **Relevant description** | **Source/Reference** |
| --- | --- | --- |
| *C. jejuni* 81-176 WT | Wild type strain | Dr. Qijing Zhang |
| *∆tlp10* | *C. jejuni* 81-176 derivative with deletion in *tlp10* gene; *tlp10*::Kan | This study |
| *∆tlp4* | *C. jejuni* 81-176 derivative with deletion in *tlp4* gene; *tlp4*::Kan | This study |
| *∆tlp6* | *C. jejuni* 81-176 derivative with deletion in *tlp6* gene; *tlp6*::Kan | This study |
| *∆tlp8* | *C. jejuni* 81-176 derivative with deletion in *tlp8* gene; *tlp8*::Kan | This study |
| *∆tlp9* | *C. jejuni* 81-176 derivative with deletion in *tlp9* gene; *tlp9*::Kan | This study |
| *tlp10comp* | *C. jejuni 81-176 tlp10 mutant* complemented with wild type copy of *tlp10* on pRY111 | This study |
| *tlp4 comp* | *C. jejuni 81-176 tlp4 mutant* complemented with wild type copy of *tlp4* on pRY111 | This study |
| *tlp6 comp* | *C. jejuni 81-176 tlp6 mutant* complemented with wild type copy of *tlp6* on pRY111 | This study |
| *tlp8 comp* | *C. jejuni 81-176 tlp8 mutant* complemented with wild type copy of *tlp8* on pRY111 | This study |
| *tlp9 comp* | *C. jejuni 81-176 tlp9 mutant* complemented with wild type copy of *tlp9* on pRY111 | This study |
| *∆cheY* (RY209) | *C. jejuni* 81-176 derivative with deletion in *cheY* gene; *cheY*::Kan | Dr. Patricia Guerry  Yao *et al.,* 1997 |
| *che2Y* (RY213) | *C. jejuni* 81-176 derivative with two copies of *cheY* gene; *astA::cheY*/Kan | Dr. Patricia Guerry  Yao *et al.,* 1997 |
| *E. coli* DH5α | *E. coli* strain used for cloning | Invitrogen |
| **Plasmids** |  |  |
| pZero-1 | Cloning vector for making suicide vector; Zeo | Invitrogen |
| pUC4K | Source plasmid for kanamycin resistance gene (Kan) | Amersham |
| pRY111 | *E.coli*-*Campylobacter* shuttle vector for complementation | Yao *et al.,*  1993 |
| pRK2013 | Helper plasmid for complementation | Akiba *et al.,* 2006 |
| pZero1-∆*tlp10* | pZero1 containing the upstream and downstream sequences of *tlp10* | This study |
| pZero1-*∆tlp10*-kan | pZero1-*tlp10* with *tlp10* gene replaced by the pUC4K kanamycin gene by inverse PCR | This study |
| pZero1-*tlp4* | pZero-1 containing the upstream and downstream sequences of *tlp4* | This study |
| pZero1-*∆tlp4*-kan | pZero1-*tlp4* with *tlp4* gene replaced by the pUC4K kanamycin gene by inverse PCR | This study |
| pZero1-*tlp6* | pZero-1 containing the upstream and downstream sequences of *tlp6* | This study |
| pZero1-*∆tlp6*-kan | pZero1-*tlp6* with *tlp6* gene replaced by the pUC4K kanamycin gene by inverse PCR | This study |
| pZero1-*tlp8* | pZero-1 containing the upstream and downstream sequences of *tlp8* | This study |
| pZero1-*∆tlp8*-kan | pZero1-*tlp8* with *tlp8* gene replaced by the pUC4K kanamycin gene by inverse PCR | This study |
| pZero1-*tlp9* | pZero-1 containing the upstream and downstream sequences of *tlp9* | This study |
| pZero1-*∆tlp9*-kan | pZero1-*tlp9* with *tlp9* gene replaced by the pUC4K kanamycin gene by inverse PCR | This study |
| pRY111-*tlp10* | pRY111 containing *tlp10* coding region for complementation; Cm^r^ (chloramphenicol resistance) | This study |
| pRY111-*tlp10* prom | pRY111 containing the putative *tlp10* promoter coding region for complementation by two step cloning; Cm^r^ | This study |
| pRY111-*tlp4* | pRY111 containing *tlp4* coding region and the upstream promoter sequence for complementation; Cm^r^ | This study |
| pRY111-*tlp6* | pRY111 containing *tlp6* coding region for complementation; Cm | This study |
| pRY111-*tlp6* prom | pRY111 containing the putative *tlp6* promoter coding region for complementation by two step cloning; Cm^r^ | This study |
| pRY111-*tlp8* | pRY111 containing *tlp8* coding region and the upstream promoter sequence for complementation; Cm^r^ | This study |
| pRY111-*tlp9* | pRY111 containing *tlp9* coding region and the upstream promoter sequence for complementation; Cm^r^ | This study |

**Table S2: Primers used in this study**

|  | Sequence |
| --- | --- |
| *Primers for gene deletion* | |
| *tlp10*F  *tlp10*R  *tlp10*F inv  *tlp10*R inv | TTAATTGGTACCTGATTTCGTTCATTTAGCCC AATTAACTGCAGAAGGTCAAAATATAGGTGGG  ATATATGGATCCTCTTCTGCTTTGTTTATGTC  TATATAGGATCCGCCTTAATGGATCTTATCCA |
| *tlp4*F  *tlp4*R  *tlp4*F inv  *tlp4*R inv | ATATATGGTACCTCAAGCATAGTATTTGCACT) TAATAACTCGAGATCTTTTCTGGAAAAAAAGG  AATTAAGGATCCGATTGATTAGCTCTTCATCG ATTTATGGATCCGGAGAACATGGTAGAGGCTT |
| *tlp6*F  *tlp6*R  *tlp6*F inv  *tlp6*R inv | TAATAAGGTACCATGATATGGGCTATACTATA ATGTATCTCGAGGATTTATTCCTCAATTTTAA  ATTATAGGATCCATATTTTTTAAAACCCCAGT TTATTAGGATCCAAAAATTAACGAGCCACATG |
| *tlp8*F  *tlp8*R  *tlp8*F inv  *tlp8*R inv | ATATATGGTACCCTCTCAAAGCTGGATAGATG ATATATCTGCAGGGATTTTAGTTCTTATCAGG  ATATATGGATCCCCCTTGATCTCTGAAAGAGA ATATATGGATCCTTTAGCTGAAAGAACAGGGC |
| *tlp9*F  *tlp9*R  *tlp9*F inv  *tlp9*R inv | AAAAAAGGTACCAGATCGCTATAAAGACTTTC AATAAACTGCAGCATTTGTGAAAAATAAAGCC  TTTAAAGAATTCGGTGTTATTAATGAGCTTAG TTTAAAGAATTCGGTGTTATTAATGAGCTTAG |
| *Complementation Primers* | |
| *tlp10* comp F  *tlp10* comp R  **tlp10* prom F  *tlp10* prom R | ATATATGGTACCTTACTGAAAGCTACTTAATT  AATTAACTGCAGAGGAGAATATCATGACAAAT  AATAATGGTACCTGGTAAATTTCTGCCTTAAT  AGATAAGGTACCATAGAAAATTTAGGAGAACT |
| *tlp4* comp F  *tlp4* comp R | TTGATTGGTACCTTAAAACCTTTTCTTCTTAA  AGTGATGTCGACGTTAATACTTGATAAAAATA |
| *tlp6* comp F  *tlp6* comp R  * *tlp6* prom F  *tlp6* prom R | ATATATGGTACCTTAATGATCTGACTCATCAA  ATATATCTGCAGCTTAGCCAATATCTAGCAAA  ATATATGGTACCTAAACTCTCCTTGTATTAGT  AATTAAGGTACCATTTTGAGGATTTATTCCTC |
| *tlp8* comp F  *tlp8* comp R | ATATATGGTACCTTATGACATCGCTTTAGCAA  AATTAACTGCAGATGGGGTTTAAGATGATTAT |
| *tlp9* comp F  *tlp9* comp R | AGTGATGGTACCTATTTTTAATTTTGCTAAGA  TGAATTCTGCAGTAAAATGACTTATAATGAAC |
| *qRT-PCR primers* | |
| *kpsM F*  *kpsM R* | CCCTAAAGCAAAAGCTGAGC  TTTGCCTATAAACCTGTAAAACCTATAC |
| *pglH F*  *pglH R* | CCTTGACATTTTCAATGCGTCC  AAACCCTTGTCATTTTAGCGATG |
| *neuB1 F*  *neuB1 R* | GTTTCAACGGGCATTGCTAC  TCCAAGTGCTACTGCCATAAC |
| *fliS F*  *fliS R* | TGCTTTATGAGGGAATTTTGCG  GAATTTCTCTTGTATAAAGCCCGC |
| *Cj0688 F*  *Cj0688 R* | GCAGTTGATTAAGCGTAGCAC  AAACAAAACGCCACAAGACG |
| *maf5 F*  *maf5 R* | GCTAGACATCTACCCTTTGCTC  CTTTCAACCTCTCCTTCTCCG |
| *luxS F*  *luxS R* | GATTTGCGTTTTTGCGTACC  CGACAGCCCATAGGTGAAAT |
| *cjj_0045 F*  *cjj_0045 R* | TTTAATCGCCTTGATACTGAT T  TTTGTGGCCAACATAGAA A |

Note: *Two step cloning was performed to create complementation plasmids for *tlp6* and *10* as they were co-transcribed with their neighboring genes with promoter regions located further upstream

**Table S3: Chemotaxis disc assay for Δ*tlp* mutants and wild type**

|  | WT | Δ*tlp10* | Δ*tlp6* | Δ*tlp9* |
| --- | --- | --- | --- | --- |
| (diameter in cm) | | | | |
| Aspartate | 3.4±0.37 | **2.2±0.25** | **2.8±0.15** | NT^c^ |
| L-Glutamine | 2.9±.033 | 2.5±0.45 | NT | NT |
| L-Serine | 4.0±0.44 | NT | NT | NT |
| Isocitrate | 2.4±0.30 | NT | **1.9±0.10** | NT |
| Fumarate | 4.1±0.26 | **3.0±0.20** | NT | NT |
| Pyruvate | 3.6±0.20 | NT | NT | 2.8±0.20 |
| Succinate | 3.2±0.26 | NT | NT | NT |
| Propionate | 2.5±0.24 | NT | NT | NT |

Note: Chemotaxis was assessed using 100 mM of each substrate by standard chemotaxis disc assay (Vegge *et al.,* 2009). Significant results (*P* < 0.05) are in bold and were determined using the student t test and by comparing wildtype values to those of each mutant independently. ^c^NT: chemical was not tested.

**References**:

Akiba, M., J. Lin, Y. W. Barton, and Q. Zhang. (2006). Interaction of CmeABC and CmeDEF in conferring antimicrobial resistance and maintaining cell viability in *Campylobacter jejuni*. *The Journal of antimicrobial chemotherapy* 57**,** 52-60.

Mazumder, R., T. J. Phelps, N. R. Krieg, and R. E. Benoit. (1999). Determining chemotactic responses by two subsurface microaerophiles using a simplified capillary assay method. *Journal of microbiological methods* 37, 255-263.

Vegge, C. S., L. Brondsted, Y. P. Li, D. D. Bang, and H. Ingmer. (2009). Energy taxis drives Campylobacter jejuni toward the most favorable conditions for growth. *Applied and environmental microbiology* 75, 5308-5314.

Yao, R., R. A. Alm, T. J. Trust, and P. Guerry. (1993). Construction of new Campylobacter cloning vectors and a new mutational cat cassette. *Gene* 130, 127-130.
