# Supplementary material for: Transducer like proteins of Campylobacter jejuni 81-176: role in chemotaxis and colonization of the chicken gastrointestinal tract
Source: Front Cell Infect Microbiol. 2015 May 27;5:46. doi: 10.3389/fcimb.2015.00046 (PMC4444964; doi:10.3389/fcimb.2015.00046)
Supplement: Supplementary file 2 [file Image1.PDF]

Figure S1

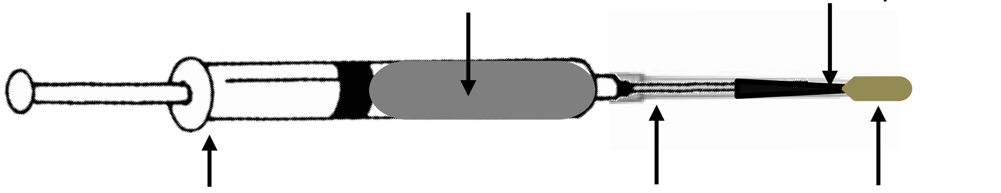

Supplemental Figure S1: The modified capillary chemotaxis apparatus to quantitatively measure bacterial tactic responses. A 100 µl of *C. jejuni* suspension was drawn into a 200 µl disposable pipette tip, which was then sealed from one end using parafilm. The needle was attached to the syringe containing the chemoattractant and the system was incubated horizontally at 42°C for 1 h. Finally, the needle-syringe system was detached and bacterial suspension in the syringe was 10-fold serially diluted in the chemotaxis buffer. Dilutions were plated onto MH agar plates, incubated for 24 h at 42°C microaerobically, and the CFU were counted.

Figure S2

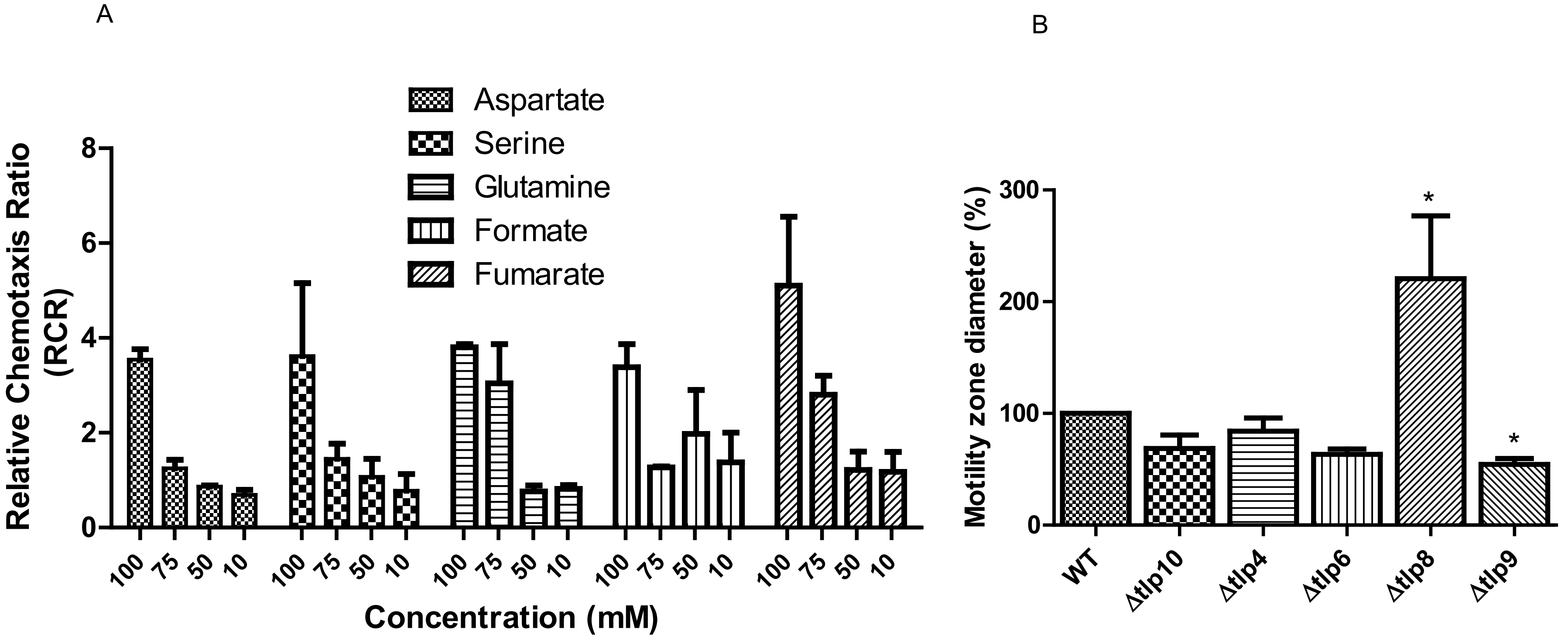

Supplemental Figure S2 A: Dose dependent chemotaxis response of wild type *C. jejuni* 81-176: Varying concentrations of chemicals ranging from 100mM to 10mM were tested for their chemotaxis towards wild type *C. jejuni* 81-176. The RCR was calculated by taking the ratio of bacterial numbers migrating towards the chemical in the syringe to the bacterial numbers migrating towards the chemotaxis buffer[4]. The results show the means and standard errors of two independent experiments. An RCR value of 2 or above indicates chemotaxis towards the test chemical. Maximum RCR values were obtained when 100mM of chemical was tested indicating that the number of bacteria migrating towards the chemical was maximum at that concentration. **B:** Histogram showing the diameter of the zone of motility in Δ*tlp* mutants, expressed in percentages, relative to the wild type.

Figure S3

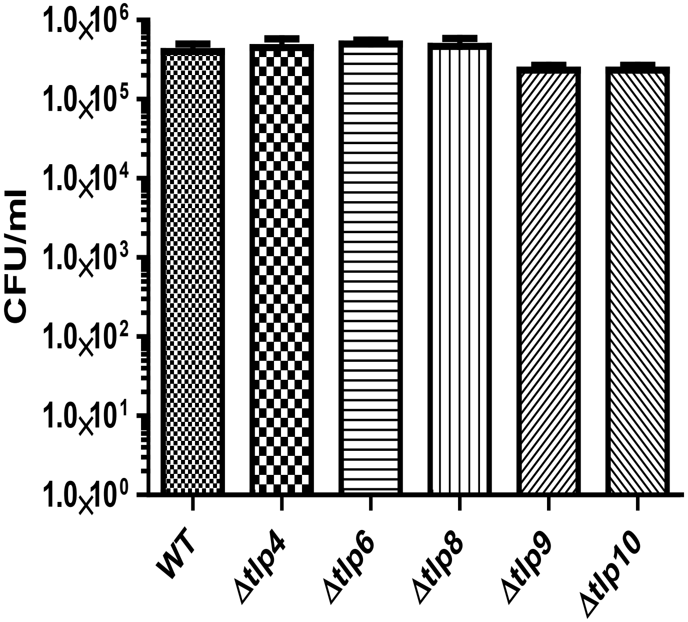

Supplemental Figure S3: Contribution of *tlps* to adherence of *C. jejuni* to INT 407 cells. INT 407 cells were infected with *C. jejuni* strains for 3 h after which the cells were washed with MEM and lysed with 0.1% Triton X-100. The resulting lysates were diluted and spread (100 µL) on MH agar plates. The data represent the average of 3 replicates in each experiment. Each bar represents the mean ± SE.

Figure S4

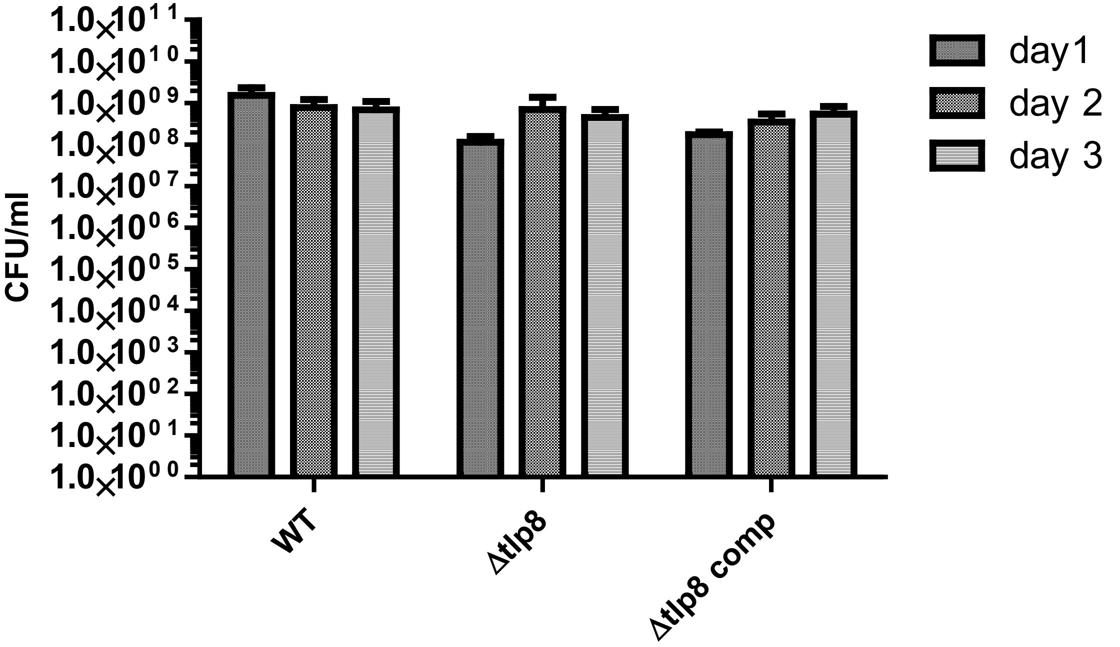

Supplemental Figure S4: Biofilm shedding on Day 1, 2 and Day 3 biofilms grown microaerobically in glass vials. Media was removed and attached biofilms were rinsed and then incubated for 1 h at 42°C in fresh MH broth. Cells shed from biofilms were collected from the supernatant after 1 h , serially diluted and plated. Colonies were counted and CFU/mL was calculated. Each vial was plated in duplicate. Each bar represents the mean ± SE of 3 independent experiments. * P≤0.05.
